# A New Miniature Characid (Ostariophysi: Characiformes: Characidae), with Phylogenetic Position Inferred from Morphological and Molecular Data

**DOI:** 10.1371/journal.pone.0052098

**Published:** 2013-01-02

**Authors:** André Luiz Netto-Ferreira, José Luís Olivan Birindelli, Leandro Melo de Sousa, Tatiane Casagrande Mariguela, Claudio Oliveira

**Affiliations:** 1 Museu de Zoologia da Universidade de São Paulo, São Paulo, São Paulo, Brazil; 2 Universidade Federal do Pará, campus Universitário de Altamira, Faculdade de Ciências Biológicas, Laboratório de Zoologia, Altamira, Pará, Brazil; 3 Departamento de Morfologia, Instituto de Biociências, Universidade Estadual Paulista, Botucatu, São Paulo, Brazil; Biodiversity Insitute of Ontario - University of Guelph, Canada

## Abstract

*Erythrocharax altipinnis* is described from the Serra do Cachimbo, Pará, Brazil. The new taxon is distinguished from all of the Characidae genera by having the pelvic bones firmly attached through the isquiatic processes; a nearly triangular hiatus in the musculature covering the anterior chamber of the swim bladder between the first and second pleural ribs (pseudotympanum); the pedunculate, notably expanded and distally compressed teeth in both jaws; circumorbital series represented by antorbital and four infraorbital bones with laterosensory canals not enclosed; a single tooth row in the premaxillary with the teeth perfectly aligned and similar in shape and cusp number; the first three branched dorsal-fin rays distinctly elongate in males; a bright red adipose and caudal fins in life; a conspicuous dark midlateral stripe extending from the opercle to the tip of the median caudal-fin rays; and by the absence of a humeral spot. The phylogenetic position of the new taxon is discussed using morphological and molecular datasets, with conflicting results of both approaches discussed. Additionally, a summarized discussion on the current problems in the Characidae taxonomy is presented and the principal biases in the morphological dataset are also discussed.

## Introduction

The family Characidae is the fourth largest family of fishes in the World, with over 1100 valid species, being only less species-rich than the freshwater Cyprinidae and Cichlidae, and the mostly marine Gobiidae [1], [2]. Contrary to these three families, the Characidae are primarily restricted to South America (ranging from Texas, U.S.A, to Argentina) and has the highest per annum rate of new species being described, 46 in 2010 [2]. Also striking in Characidae is the poor knowledge about the taxonomy of the species and their interrelationships. Approximately half of the entire diversity of the Characidae was listed as *incertae sedis* by Lima et al. [3]. In addition, most of the larger genera, such as *Astyanax* Baird & Girard, *Moenkhausia* Eigenmann, *Hyphessobrycon* Durbin, and *Hemigrammus* Gill, are considered to be non-monophyletic [3], and to require in depth analyses [1], due to species-level taxonomic problems.

Recently, Mirande [4] proposed a new hypothesis of relationships within the Characidae based on morphological characters. His analysis incorporated a large number of taxa and characters, and used implied weighting to achieve a better resolution. The major outcomes of his analysis were to define a monophyletic Characidae, composed of 15 monophyletic subfamilies and 7 unnamed monophyletic clades, as well as the corroboration of the non-monophyly of the largest *incertae sedis* genera as proposed by Lima et al. [3]. Concomitant with Mirande [4], and succeeding a series of anterior studies focusing in the relationships of the Characiformes [5], [6], [7], two important papers were recently published using molecular data to investigate the phylogenetic relationships within the Characidae [8], [9]. Both contributions proposed similar relationships among the Characidae, but Oliveira et al [9], the most comprehensive of these studies, proposed a more constrained Characidae composed of four clades, one consisting exclusively of *Spintherobolus*, the second being the Stervadiinae (similar to that proposed by Mirande [4] but with the inclusion of *Markianna*), the third comprising the Aphyocharacinae, Characinae, Cheirodontinae and Tetragonopterinae, and the fourth including Stethaprioninae, Rhoadsiinae and a variety of genera, most previously recognized as *incertae sedis* by Lima et al. [3].

A recent expedition to the headwaters of the rio Curuá yielded a new species of characid, which could not be allocated to any described genus. The aim of the present contribution is to describe this new taxon and discuss the different hypotheses of its phylogenetic placement within Characidae based on both morphological and molecular data using the frameworks of the aforementioned, recently published, studies focused in the relationships within the family.

## Materials and Methods

Counts and measurements follow Fink & Weitzman [10] and Menezes & Weitzman [11]. All measurements were made point-to-point with dial calipers and data recorded to tenths of a millimeter on the left side of the specimens whenever possible. Standard length is presented in mm, all other measurements are presented as proportions of standard length, except subunits of head, presented as proportions of head length. Meristic data are given in the description, followed by the frequency for each count in parenthesis and an asterisk indicating counts of the holotype. Vertebrae, supraneurals, procurrent caudal-fin rays, branchiostegal rays, gill-rakers, teeth counts and teeth cusps number were taken only from cleared and double-stained paratypes (c&s) prepared according to Taylor and Van Dyke [12]. Vertebrae of the Weberian apparatus were included as four elements, and the fused PU1+U1 of the caudal region as a single element. Pattern of circuli and radii was observed on scales sampled from the region between the lateral line and the insertion of the dorsal fin. Specimens were collected under permit number 104/2006 issued by DIFAP/IBAMA. The collecting locations reported in this work were not protected in any way and the field studies did not involve endangered or protected species. In the material listed, the specimens are all preserved in alcohol, except when cleared and stained, indicated by CS. Institutional abbreviations are ANSP for Academy of Natural Sciences of Philadelphia, LBP for Laboratório de Biologia e Genética de Peixes, and MZUSP for Museu de Zoologia da Universidade de São Paulo.

The morphologic phylogenetic study follows Mirande's [4] framework with the posterior additions of Mirande et al. [13] and Malabarba et al. [14], whereas the molecular phylogeny methodology (data production and analysis) is based in Oliveira et al. [9] and the results of Tagliacollo et al. [15] and Mariguela et al. [16]. The cladistic analysis of the morphological dataset was performed with aid of the software Tree analysis using New Technology – TNT, licensed to the Willi Hennig Society [17] using the heuristic search algorithim “Mult” with 5000 replicates, using the TBR branch swap algorithm. Fundamental trees obtained were submited to an additional branch swap by the command “bb” to search for unexplored islands of trees with less steps. Characters regarding bones or associated structures absent in *Erythrocharax* (i.e. laterosensory canal system of absent infraorbitals; modifications of the rhinosphenoid) were coded as inapplicable, contrary to Mirande [4], who attributed states to characters fitting these conditions. The complete coding for *Erythrocharax altipinnis* following character sequence presented in Mirande [4] and subsequently modified by Mirande et al. [13] is presented in Table S1. All sequences obtained were deposited in GenBank under the accession numbers: 16S: JX570761, CytB: JX570762, Myh6: JX570763, Rag2: JX570764.

### Nomenclatural Acts

The electronic edition of this article conforms to the requirements of the amended International Code of Zoological Nomenclature, and hence the new names contained herein are available under that Code from the electronic edition of this article. This published work and the nomenclatural acts it contains have been registered in ZooBank, the online registration system for the ICZN. The ZooBank LSIDs (Life Science Identifiers) can be resolved and the associated information viewed through any standard web browser by appending the LSID to the prefix “http://zoobank.org/”. The LSID for this publication is: urn:lsid:zoobank.org:pub:2E486AD8-5387-4EA5-B744- 7B4DD34A79FA. The electronic edition of this work was published in a journal with an ISSN, and has been archived and is available from the following digital repositories: PubMed Central, LOCKSS.

## Results

### Erythrocharax, new genus

(urn:lsid:zoobank.org:act:525F06FB-CF25-482B-9E26-6B34EF3380F6)

### Figs. 1A–B, 2

**Figure 1:**
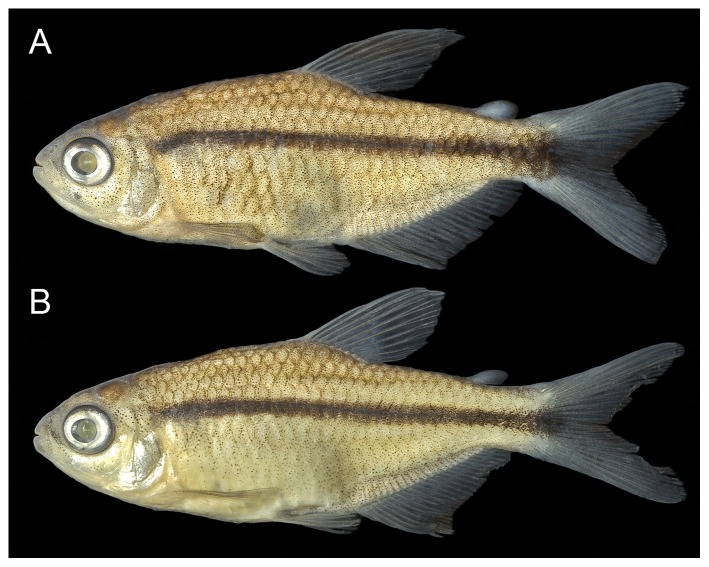
*Erythrocharax altipinnis*. (a) holotype, MZUSP 111000, 26.2 mm SL, (b) paratype, MZUSP 110999, 22.4 mm SL, rio Curuá, tributary of rio Iriri, rio Xingu basin.

**Figure 2:**
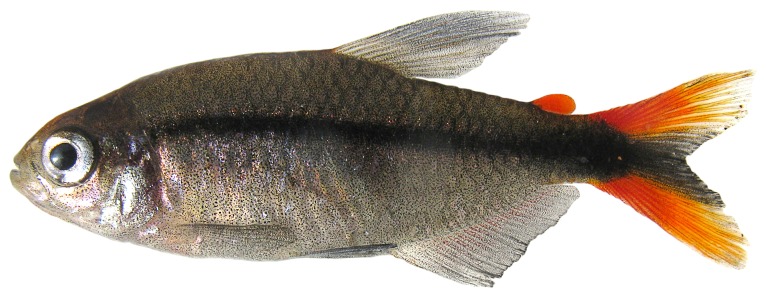
*Erythrocharax altipinnis* live paratype, MZUSP 110999, 25.8 mm SL, rio Curuá, tributary of rio Iriri, rio Xingu basin, depicting live color pattern of species.

#### Type species

*Erythrocharax altipinnis*, new species, by monotypy and original designation.

#### Diagnosis

**Figure 3:**
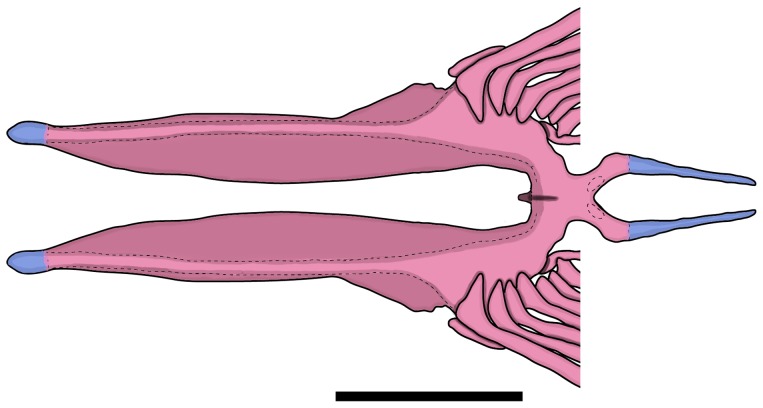
Dorsal view of pelvic girdle of *Erythrocharax altipinnis*, MZUSP 110999, 23.5 mm SL. Scale bar = 1 mm.

**Figure 4:**
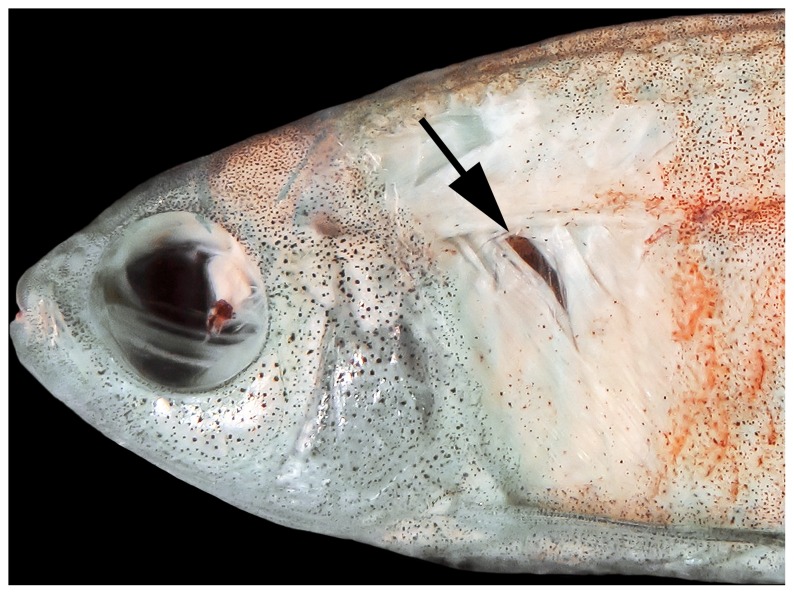
Lateral view of dissected paratype of *Erythrocharax altipinnis* MZUSP 110999, 23.5 mm SL, depicting the pseudotympanum (indicated by arrow).

**Figure 5:**
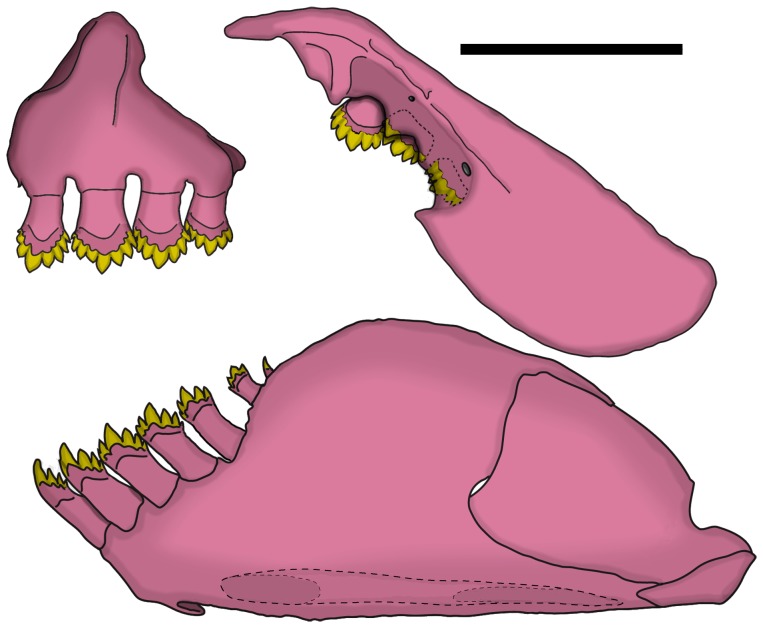
Lateral view of upper and lower jaws of *Erythrocharax altipinnis*, MZUSP 110999, 23.5 mm SL. Scale bar = 1 mm.

**Figure 6:**
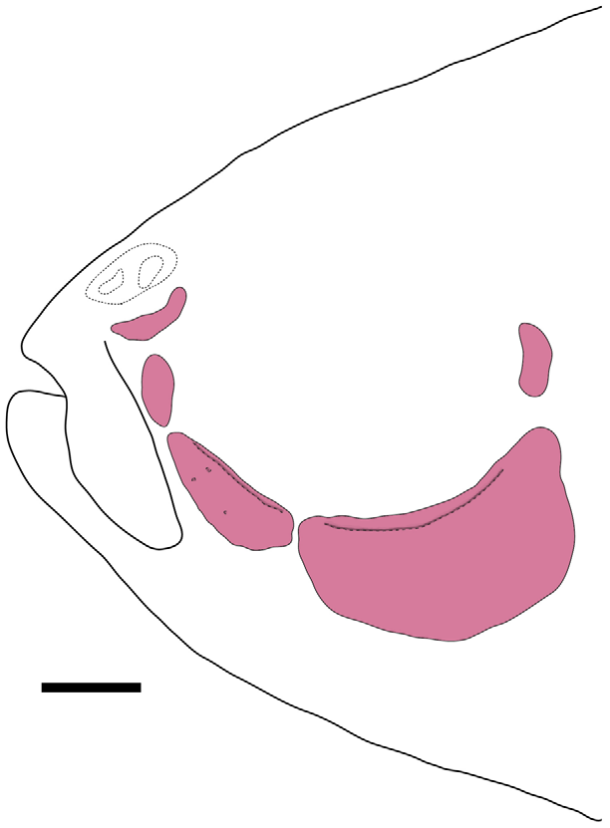
Circumorbital series of *Erythrocharax altipinnis*, MZUSP 110999, 23.5 mm SL. Scale bar = 1 mm.

*Erythrocharax* is distinguished from all other genera of the Characidae by having the pelvic bones firmly attached through the isquiatic processes (Fig. 3). Additionally, the new genus can be further distinguished from other Characidae by having a nearly triangular hiatus in the musculature covering the anterior chamber of the swim bladder between the first and second pleural ribs (pseudotympanum – Fig. 4); the pedunculate, notably expanded and distally compressed teeth in both jaws (Fig. 5); circumorbital series represented by antorbital and four infraorbital bones with laterosensory canals not enclosed (Fig. 6); a single tooth row in the premaxillary with the teeth perfectly aligned and similar in shape and cusp number; the first three branched dorsal-fin rays distinctly elongate in males; a bright red adipose and caudal fins in life; a conspicuous dark midlateral stripe extending from the opercle to the tip of the median caudal-fin rays; and by the absence of a humeral spot.

#### Etymology

From the Greek *erythrus*, meaning red, in reference to the bright red coloration of the adipose and caudal fin in live specimens, plus the suffix *-charax*, as generally applied for genera of the Characidae.

### *Erythrocharax altipinnis*, new species

(urn:lsid:zoobank.org:act:AF75AED3-B651-416C-B3CB-64410807DA6D)

### Figs. 1A–B, 2

#### Holotype

MZUSP 111000 (26.2 mm SL), Brazil, Pará, rio Curuá, tributary of rio Iriri, on bridge of BR-163, 8°53′54″S 54°59′20″W, rio Xingu Basin, 22 Jan 2009, J. L. Birindelli, A. L. Netto-Ferreira, L. M. Sousa & P. Hollanda-Carvalho.

#### Paratypes

ANSP 199143 (1, 24.3 mm SL), MZUSP 110999 (6, 22.4–26.0 mm SL, 1 CS, 23.5 mm SL), LBP 10881 (1, 24.3 mm SL): collected with holotype.

#### Diagnosis

Same as that of the genus.

#### Description

Morphometric data presented in Table 1. Overall size small (largest examined specimen 26.2 mm SL). Body compressed, moderately elongate. Greatest body depth at dorsal-fin origin. Dorsal profile of head slightly convex from upper lip to vertical through nares; mostly straight from latter point to tip of supraoccipital spine; convex from tip of supraoccipital spine to dorsal-fin origin, straight from dorsal-fin base to adipose fin; slightly concave between latter point and origin of anteriormost dorsal procurrent caudal-fin ray. Ventral profile of head and body distinctly convex from lower lip to anal-fin origin; slightly convex along anal-fin base, and concave between terminus of anal-fin and anteriormost procurrent caudal-fin ray. Circumorbital series represented by antorbital and four infraorbital bones (Fig. 6). Relatively large, nearly triangular hiatus in muscles covering laterally anterior chamber of swim bladder between first and second pleural ribs (pseudotympanum).

**Table 1:** Morphometric data for *Erythrocharax altipinnis*.including holotype and eight paratypes.

	Holotype	N	Mean	Range			SD
Standard Length (mm)	26.2	9	24.1	22.4	-	26.2	
**Percentages of Standard Length**							
Depth at dorsal-fin origin	34.7	9	34.4	33.1	-	36.0	0.98
Snout to dorsal-fin origin	55.8	9	54.8	53.3	-	55.8	1.01
Snout to pectoral-fin origin	29.8	9	29.8	28.4	-	31.1	0.89
Snout to pelvic-fin origin	47.9	9	48.2	44.2	-	51.0	1.90
Snout to anal-fin origin	65.5	9	64.4	62.4	-	66.8	1.43
Caudal-peduncle depth	10.5	9	11.3	10.5	-	11.7	0.45
Caudal peduncle length	8.97	9	9.2	7.8	-	10.5	0.87
Pectoral-fin length	20.0	9	19.4	17.8	-	20.7	1.13
Pelvic-fin length	17.8	9	17.9	14.9	-	20.5	1.97
Dorsal-fin base length	14.5	9	14.4	13.1	-	15.8	0.95
Dorsal-fin depth	32.9	9	32.4	30.2	-	35.7	2.17
Anal-fin base length	32.1	9	30.5	28.3	-	33.6	1.67
Anal-fin lobe length	19.6	9	18.6	17.8	-	19.6	0.53
Eye to dorsal-fin origin	40.7	9	41.0	39.9	-	42.0	0.74
Dorsal-fin origin to caudal-fin base	53.0	9	51.7	49.9	-	53.0	1.04
Bony head length	27.6	9	28.2	26.8	-	30.3	1.00
**Percentages of head length**							
Horizontal eye diameter	37.5	9	35.4	33.2	-	37.5	1.42
Snout length	23.0	9	24.1	20.3	-	27.1	2.13
Least interorbital distance	27.7	9	26.2	22.5	-	29.5	2.08
Upper jaw length	31.1	9	30.9	26.9	-	34.8	2.09

SD – Standard Deviation.

Mouth terminal. Rear terminus of maxilla reaching vertical through anterior margin of orbit or slightly beyond that part. Premaxillary teeth in single row of four*(9) pedunculate, distally compressed, hexa-, hepta- or octacuspid teeth (Fig. 5). Maxilla with three*(6) pedunculate, distally compressed, hexa- or heptacuspid teeth decreasing in size posteriorly. Dentary with seven (1) pedunculate, distally compressed teeth; anteriormost four teeth largest, hexa- or heptacuspid; two subsequent teeth pentacuspid, and posteriormost tooth conic, all distinctly smaller than four anteriormost teeth. First gill arch with 2(1) hypobranchial, 7(1) ceratobranchial, 1(1) on cartilage between ceratobranchial and epibranchial, and 5(1) epibranchial gill-rakers. Branchiostegal rays 4(1), three originating on anterior ceratohyal and one on posterior ceratohyal.

Scales cycloid, circuli absent on exposed area of scales, radii absent. Lateral line lon with 31(2), 32*(3) or 33(4) scales, 5*(4), 6(4) or 7(1) of which perforated. Horizontal scale rows between dorsal-fin origin and lateral line 5(3) or 6*(6). Horizontal scale rows between lateral line and pelvic-fin insertion 5*(9). Predorsal scales 10(1), 11*(6) or 12(2). Single row of 3(3), 4*(5) scales covering base of anteriormost anal-fin rays. Circumpeduncular scales 14*(9). Caudal fin with scales only at base of both lobes.

Pectoral-fin rays i,10(4), 11*(4) or 12(1). Tip of pectoral fin reaching vertical through pelvic-fin origin. Pelvic-fin rays i,6*(9). Supraneurals 6(1), situated anterior of neural spine of 5^th^ to 10^th^ centra. Dorsal-fin rays ii, 9*(9). Dorsal-fin origin slightly posterior to middle of SL. Base of posteriormost dorsal-fin ray posterior to vertical through anal-fin origin. First dorsal-fin pterygiophore inserted posterior of neural spine of 10^th^(1) centrum. Adipose fin present. Anal-fin rays iv(1), 20(2), 21*(5) or 22(2). Anteriormost anal-fin pterygiophore inserted behind haemal spine of 17^th^(1) centrum. Caudal fin forked, lobes slightly rounded and similar in size. Principal caudal-fin rays i,9/i,7*(9). Dorsal procurrent caudal-fin rays 10(1); ventral procurrent caudal-fin rays 7(1). Total vertebrae 34(1) with 16(1) precaudal and 18(1) caudal.

#### Color in alcohol

Ground light yellowish with chromatophores lightly scattered over entire body in males, and all except ventral portion in females. Lower lip, snout, top of head and dorsal portion of body lightly pigmented, resulting in weak countershaded color pattern. Humeral blotch absent. Five dorsalmost longitudinal scale rows with dark chromatophores along distal border of scales, forming discrete reticulate pattern in mid-dorsal portion of dorsolateral surface. Narrow, conspicuous, dark midlateral stripe formed by concentrated dark chromatophores along longitudinal septum of body slightly curved ventrally anteriorly, extending from pectoral girdle to tip of caudal peduncle, becoming distinctly wider from the latter to tip of median caudal-fin rays. All fins somewhat hyaline, with distinct concentration of chromatophores on rays and intervening membranes (Fig. 1A–B).

#### Color in life

Ground color of body grayish, with blood red adipose, upper and lower caudal-fin lobes (Fig. 2).

#### Sexual dimorphism

Despite their minute size (22.4–26.2 mm SL), specimens of *Erythrocharax* examined herein were found to be mature and sexually dimorphic, representing a miniature species in the sense of Weitzman & Vari [18]. Males of *Erythrocharax* have distinctly darker background coloration of the body with the longitudinal stripe wider and more intense coloration of fins (Fig. 1A). Additionally, adult males also have distinctly longer dorsal- and anal-fin rays, forming a nearly straight distal margin (vs. anal-fin distal margin concave in females – Fig. 1A and 1B, respectively). Bony hooks were not observed on the fin rays.

#### Distribution

**Figure 7:**
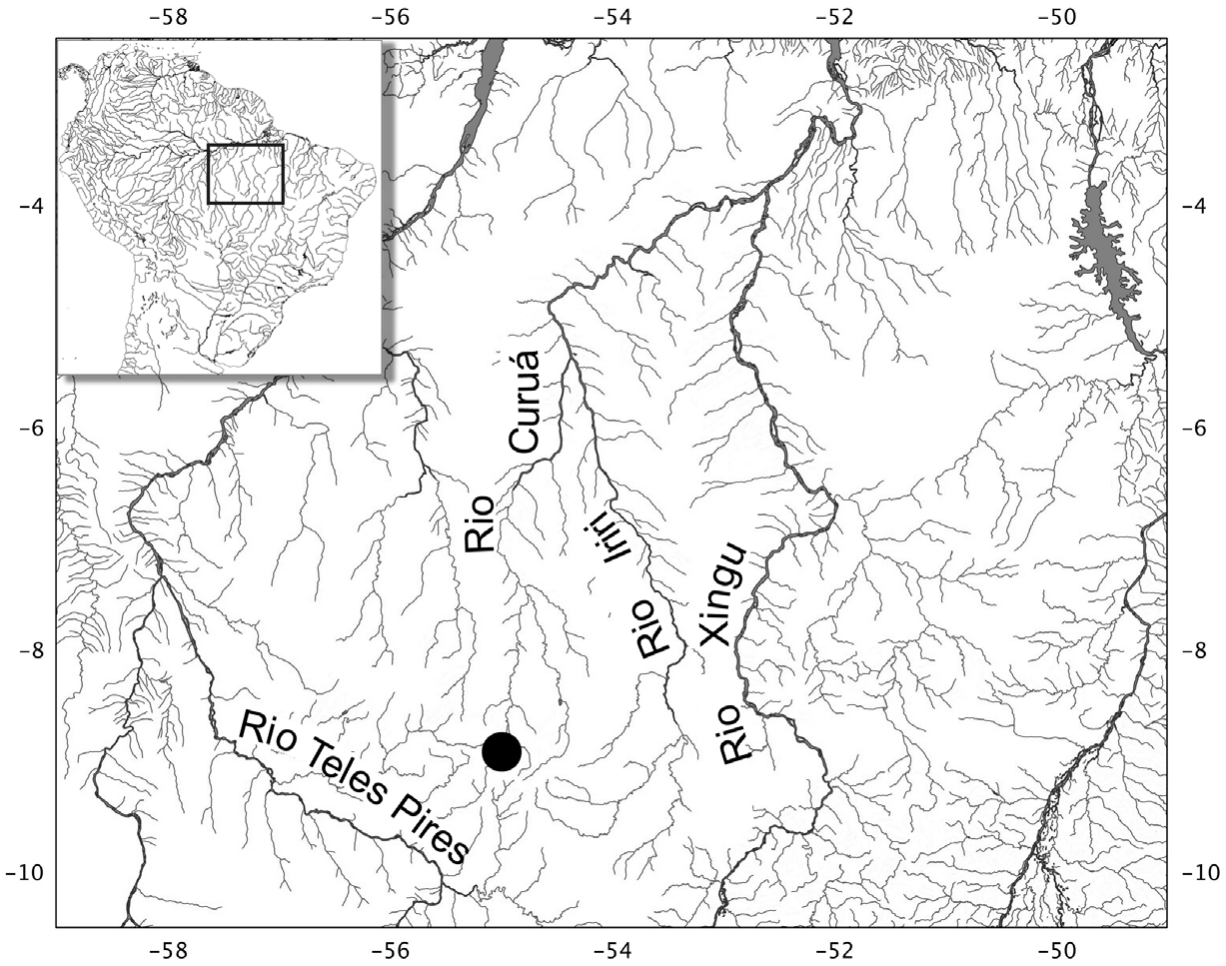
Map of Serra do Cachimbo and adjoining areas, showing distribution of *Erythrocharax altipinnis*.

**Figure 8:**
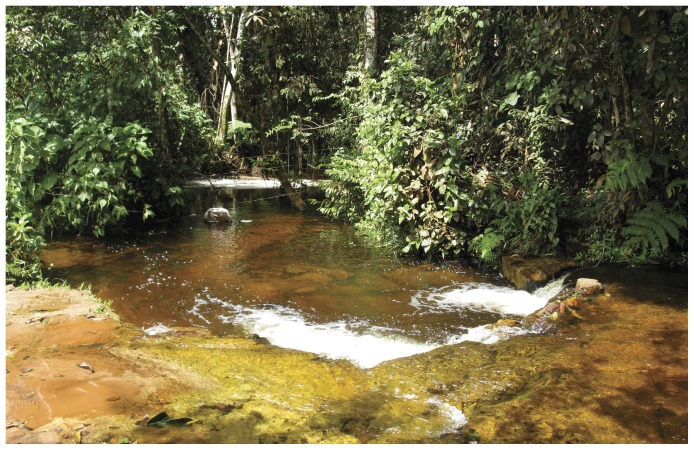
Type locality of *Erythrocharax altipinnis*. Rio Curuá, tributary of rio Iriri, on bridge of BR-163 (8°53′54″S 54°59′20″W), rio Xingu Basin.

The new species is only known from its type locality (Figs. 7 and 8), the rio Curuá, at Serra do Cachimbo, Pará, Brazil.

#### Etymology

From the latin *alti*, meaning elongate, and *pinnis* meaning fin, in reference to the elongate dorsal-fin rays in males.

## Discussion

The presence of pelvic bones firmly attached through the isquiatic processes seem to represent a unique condition among the Characidae sensu Oliveira et al. [9]. Similar condition was described by Mirande [4] in the triportheids *Agoniates* Müller & Troschel, *Clupeacharax* Pearson, and *Engraulisoma* Castro, where the pelvic bones also articulate. That condition differs from the observed in *Erythrocharax*, in which the pelvic bones are strongly ankylosed along most of its medial surface, with only a discrete medio-sagittal septum in its ventral surface, whereas in those members of the Triportheidae the pelvic bones articulate via interdigitations of the ischiatic processes which corrugate the counterparts [4]. The remaining diagnostic characters of *Erythrocharax*, on the other hand, seem to have complex evolution patterns in Characidae and have apparently evolved and reversed independently several times in the family as discussed in [4], [14], [19], [20]. The pseudotympanum, for example, is a feature present in several subsets of Characidae and its distribution in the family has been exhaustively discussed by several authors [21], [22]. Similarly, the presence of a single row of teeth in the premaxillary represents the plesiomorphic condition of the Characiformes and is also a widespread feature in Characidae. However, among all the taxa with a single premaxillary tooth row, only *Erythrocharax*, *Coptobrycon*, *Spintherobolus* and the Cheirodontinae (except *Prodontocharax*) present multicuspid, largely expanded, distally compressed teeth, whereas all other groups present conical or tricuspid cylindrical teeth. *Erythrocharax* can be distinguished from *Coptobrycon* by the absence of a longitudinal stripe onto the anal-fin base, and the presence of maxillary teeth (vs. longitudinal stripe present and maxillary teeth absent [23]), and from *Spintherobolus* by lacking the anteriormost proximal radial of the anal fin with an anteriorly extended lamina, and short caudal peduncle (vs. anteriormost proximal radial with anteriorly extended lamina and caudal peduncle extremely elongate). Although *Erythrocharax* has all the synapomorphic features listed by Malabarba [21] defining the Cheirodontinae, it is distinguished from all genera of that group with teeth bearing spatulated crowns by the presence of distinctly separate nostrils (vs. anterior and posterior nostrils overlapping, separated only by a skin fold). *Erythrocharax* is further distinguished from all the Cheirodontini by lacking modifications of the procurrent caudal-fin rays [21]; from the Compsurini by lacking modifications on rays, scales and soft tissues of the caudal fin [21]; from *Odontostilbe* by having the second unbranched dorsal-fin ray and the unbranched pelvic-fin ray not elongated; from *Pseudocheirodon* by having the symphyseal dentary joint articulated through intercalated bony folds, the posterior tip of maxilla wide, and the maxilla straight (vs. curved); from *Aphyocheirodon* and *Cheirodontops* by having dentary teeth with seven cusps of variable size, forming a distinctly convex edge (vs. three central cusps equally sized and forming a nearly straight edge); from *Acinocheirodon* by lacking ventral expansions of ventral procurrent caudal-fin rays, and lacking modifications on rays of ventral lobe [24]; from *Kolpotocheirodon* by lacking a specialized caudal organ; from *Ctenocheirodon* by presenting seven ventral caudal-fin procurrent rays, not forming a ventral keel along ventral margin of caudal peduncle of male specimens (vs. 16–19 procurrent rays forming a keel on males [25]).

Despite the morphological similarities between *Erythrocharax* and the aforementioned genera and the Cheirodontinae of Malabarba [21], phylogenetic analyses including the new taxon in Mirande's [4] data matrix (with additions of Mirande et al. [13], and Malabarba et al. [14]) results in the genus being deeply nested in the Characidae of Mirande [4], and included with Rhoadsiinae, as the sister group of a clade comprised by (Cheirodontinae+(Aphyoditeinae+(Aphyocharacinae+Stevardiinae))) (complete tree available as Figure S1). This group is supported by five synapomorphies common to all fundamental trees obtained in the traditional search with equal weights: the sphenotic spine not extending ventrally to the articulation between the sphenotic and hyomandibula (Char. 10); the short mesethmoid spine, with the premaxillae articulating with each other anterior to the mesethmoid (Char. 27); the maxilla not reaching posterior margin of Meckel's cartilage (Char 100); a single row of premaxillary teeth (Char. 122 – secondarily reverted in Stevardiinae); two rows of teeth of gill rakers on the second ceratobranchial (Char. 193); and the presence of a pseudotympanum (Char. 339 – secondarily reverted in Stevardiinae). The search using implied weights with K values ranging from 0–21 resulted in the same relationships for *Erythrocharax*, Rhoadsiinae, and the clade containing (Cheirodontinae+(Aphyoditeinae+(Aphyocharacinae+Stevardiinae)). The results of all analyses strongly differ from the relationship hypothesis of several taxa included in clade 202 of Mirande [4] (Figure S1).

In the maximum likelihood analysis based on molecular data, *Erythrocharax* falls within the clade C of Oliveira et al. [9] as the sister group of *Parecbasis cyclolepis* plus ‘*Macropsobrycon’ xinguensis* (complete tree available as Figure S2). *Parecbasis cyclolepis* and ‘*Macropsobrycon’ xinguensis* seem to be closely related based on several morphological features, such as the upturned mouth with a single row of tricuspid teeth on both jaws, and the presence of a dark blotch at the base of the median caudal-fin rays. Alternatively, *Erythrocharax altipinnis* is clearly distinguished from these taxa in lacking a caudal blotch and bearing multicuspid pedunculate teeth with spatulate crowns.

Thus, the new taxon described herein presents three distinct alternative hypotheses of relationships: one based on the presence of the diagnostic characters of the Cheirodontinae as proposed by Malabarba [21]; the second based on morphological characters following Mirande [4] and Mirande et al. [13]; and the third based on molecular data [9]. Therefore, the exclusion of *Erythrocharax* from any recognized genus in Characidae by all three approaches, as well as the definitions of the genera following classical studies such as those of Eigenmann [26], underpins the current decision to erect a new name for the described taxon.

Recently, several authors have debated the use of different sources of characters (especially morphological and molecular data) in phylogenetic analyses [27]. Although our contribution primarily deals with the description of a new monotypic genus, the attempt to understand its relationships using the three alternative approaches shows, quite obviously, that the definition and understanding of relationships among species of Characidae is still incomplete in Neotropical Ichthyology, and lacks robust data. Such robustness should not only include the use of advanced methodological approaches to reduce biased topologies, but also extended towards taxa and character sampling, in an attempt to produce more stable phylogenetic hypotheses. At this time, ichthyologists stand at a point where less than 20% of the characid diversity has been included in any morphological and/or molecular analyses [4], [8], [9], [13], and these studies do not represent the complete picture of relationships within the family.

In addition to the concerns involving effects of the subsampling on clade stability, and therefore, nomenclature stability, it is also important to highlight problems regarding the current morphological framework of the Characidae phylogeny. Mirande [19] proposed a criterion termed “order”, for selecting the “K” (concavity constant) in analyses using implied weighting searches. However, Mirande [19] was not explicit as how to calculate the order either in that or subsequent contributions [4], [13] in which the criterion was applied. Details as to the criteria employed to establish the limits of high or low “order” are also lacking, making it impossible to replicate similar criteria for the phylogenetic analyses using a matrix modified from Mirande's data. Not only is this problematic in terms of the Scientific Method, but replicability of previous analytical settings is crucial for clade stability in future phylogenetic studies of the Characidae, since several clades defined in the hypotheses presented by Mirande [4] have rather low support and the use of distinct “K” values greatly changes the relationships among several species, genera and subfamilies. Some of Mirande's [4] characters must also be submitted to further scrutiny regarding homology criteria used to propose characters such as “Presence of bony hooks”, in which hooks coded as present regardless of which fin bears such hooks. Although, the presence of bony hooks on fins rays is a widespread feature of the Characidae, the homology of these structures depend on their shape, position, and distribution in each fin. Mirande [4] split continuous values of meristic features in several dependent binary characters, such as those involving (e.g., fin ray numbers; number of teeth), or those involving even presence/absence and distribution of bony hooks (characters 307–316), resulting in excessive weight to the presence in regard to absence of such structures. Additionally, several characters of Mirande [4] have problematic state definitions, with state descriptions not complementing each other, leaving an “intermediate zone” between them (i.e. characters 137, 219, 226). In such cases Mirande [4] coded the intermediate forms as polymorphic instead of assigning new states to those characters. Both cases affect the primary requisite of character and character state proposition: characters should be independent among each other; and character states should be mutually exclusive [28], and, therefore, complementary. Finally, the phylogenetic significance of characters such as “angle between the lateral ethmoids” (character 16) or “number of 2n chromosomes” (character 361–365), among others, must also be critically examined. Such problems in the current morphological dataset along with the subsample problems discussed above demand careful review and should not be overlooked in future contributions so that a more stable classification of Characidae can be achieved.

## Supporting Information

Table S1 Character states of *Erythrocharax altipinnis*, gen. n. and sp. n. Character follows that of Mirande [4], with the addition of Mirande et al. [13].
(DOC)

Figure S1 **Phylogenetic relationships among the Characidae constructed with morphological data.** The position of *Erythrocharax altipinnis* is shown. Incongruences between morphological and molecular approaches, and shared terminal taxa are demonstrated through colored clades matching colors presented in the Maximum Likelihood tree (Figure S2).
(TIF)

Figure S2 **Phylogenetic tree showing relationships among the characid species analyzed by a Maximum Likelihood (ML) partitioned analysis of the concatenated dataset.** The numbers at each node represents the percentage of bootstrap support obtained by ML (1000 bootstrap replicates). Nodes not supported by values higher than 50% were collapsed. The position of *Erythrocharax altipinnis* in the Clade C is shown in red.
(TIF)
