# Supplementary material for: A New Miniature Characid (Ostariophysi: Characiformes: Characidae), with Phylogenetic Position Inferred from Morphological and Molecular Data
Source: PLoS One. 2013 Jan 2;8(1):e52098. doi: 10.1371/journal.pone.0052098 (PMC3534666; doi:10.1371/journal.pone.0052098)
Supplement: Table S1 — Character states of Erythrocharax altipinnis, gen. n. and sp. n. Character follows that of Mirande [4], with the addition of Mirande et al. [13]. (DOC) [file pone.0052098.s001.doc]

**Table S1.** Character states of *Erythrocharax altipinnis*, gen. n. and sp. n. Character follows that of Mirande [4], with the addition of Mirande et al. [14].

*Erythrocharax altipinnis*

00010010-0 0011001000 1001101001 0100100101 0001010--- 0100000100 0-100----1

-01----010 0-11--01-- 10-1101001 0-11000010 1000000110 0000?11100 0001100110

001?000001 ?010001000 0010000201 1001--0110 0101100101 0111110000 1?11000010

0100000101 0100000100 1000000000 0000000001 0101000100 1001110101 1000010001

0000111000 0000010101 0000000??? ??????0010 --00000010 01?1011010 000000000?

1000000??? ?????0
