# Supplementary figures and images for: A New Miniature Characid (Ostariophysi: Characiformes: Characidae), with Phylogenetic Position Inferred from Morphological and Molecular Data
Source: PLoS One. 2013 Jan 2;8(1):e52098. doi: 10.1371/journal.pone.0052098 (PMC3534666; doi:10.1371/journal.pone.0052098)

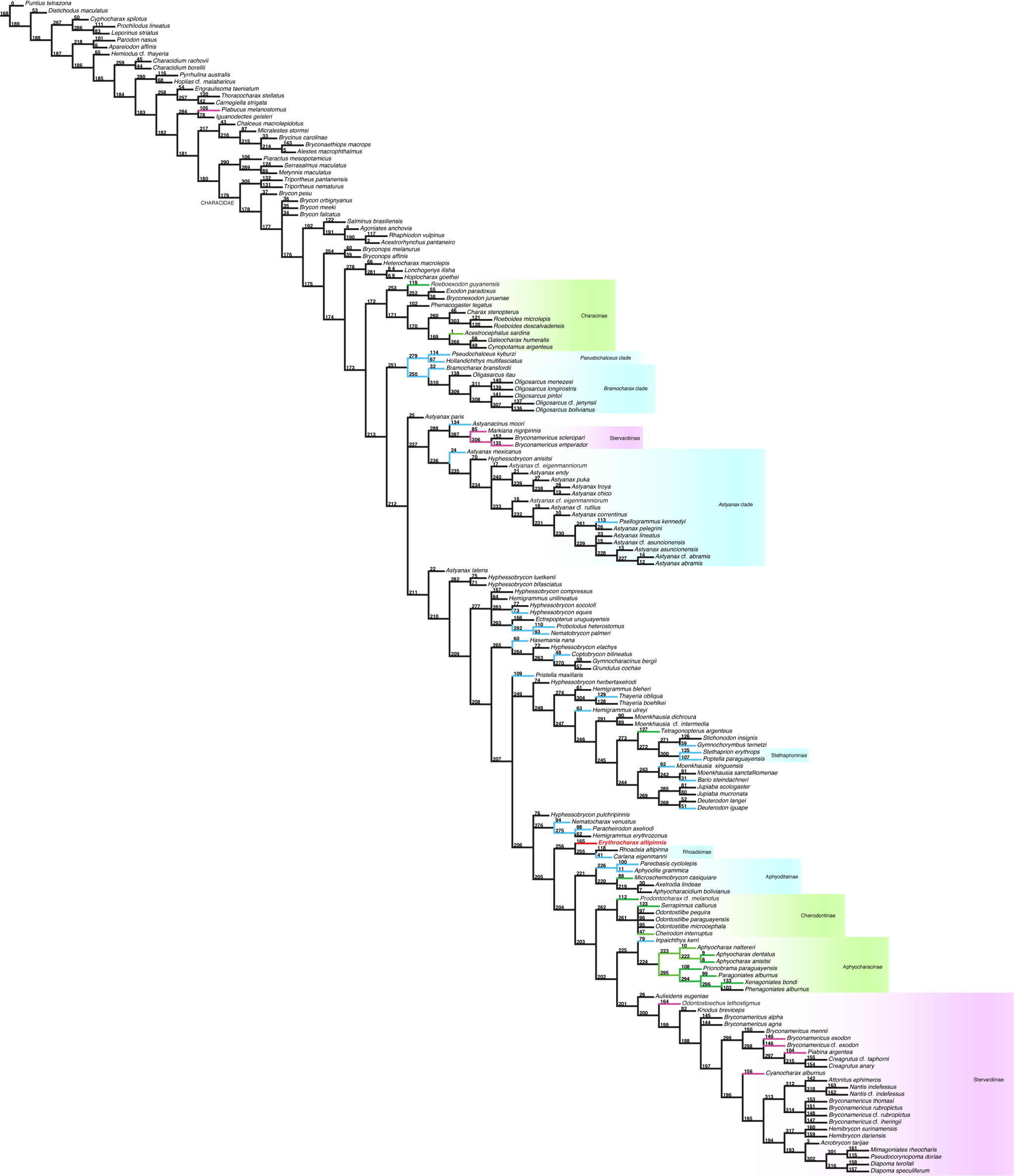

Supplement: Figure S1 — Phylogenetic relationships among the Characidae constructed with morphological data. The position of Erythrocharax altipinnis is shown. Incongruences between morphological and molecular approaches, and shared terminal taxa are demonstrated through colored clades matching colors presented in the Maximum Likelihood tree (Figure S2). (TIF) [file pone.0052098.s002.tif]

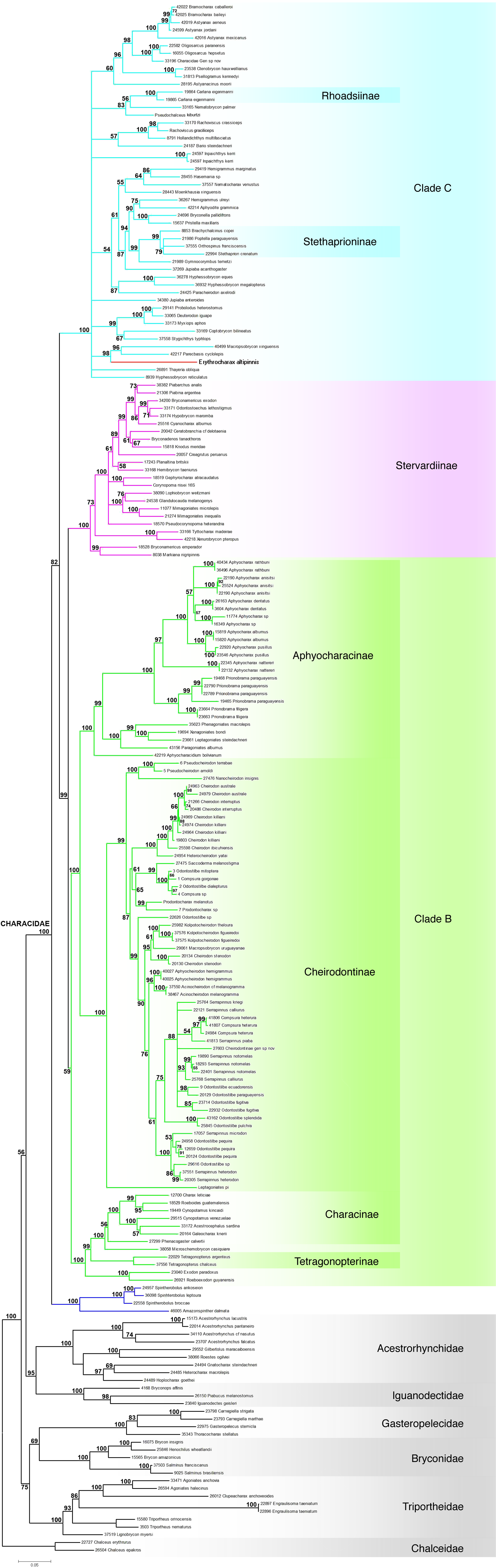

Supplement: Figure S2 — Phylogenetic tree showing relationships among the characid species analyzed by a Maximum Likelihood (ML) partitioned analysis of the concatenated dataset. The numbers at each node represents the percentage of bootstrap support obtained by ML (1000 bootstrap replicates). Nodes not supported by values higher than 50% were collapsed. The position of Erythrocharax altipinnis in the Clade C is shown in red. (TIF) [file pone.0052098.s003.tif]
